# Inflammatory Pseudotumor of the Liver with *Escherichia coli* in the Sputum

**DOI:** 10.1155/2015/249210

**Published:** 2015-08-12

**Authors:** Sreenath Narayan, Ashwini Nayak, Chris L. King

**Affiliations:** Case Western Reserve University School of Medicine, Louis Stokes Cleveland VA Medical Center, Cleveland, OH 44106, USA

## Abstract

Inflammatory pseudotumor is a nonmalignant lesion that mimics malignant lesions and has been reported to occur at various sites throughout the body. Though it has been reported as a reaction to infection, the true etiology of the lesion is unknown. In this report, we present the case of a patient with a liver lesion of unknown origin. Through a series of imaging studies, we were able to observe the locally aggressive nature of this lesion as it rapidly eroded into the lung. Sputum cultures showed growth of *E. coli*, indicating *E. coli* infection as a possible etiology of this lesion. Pathology was consistent with inflammatory pseudotumor.

## 1. Introduction

Inflammatory pseudotumor (IPT) is a rare, nonmalignant lesion that can present a diagnostic challenge because of its ability to clinically and radiographically mimic malignant processes [1–3]. However, IPT lesions are not malignant, and it is important to distinguish them from malignant processes for treatment and prognostic purposes.

The lungs are the most common locations for IPT [1], followed by the liver [4]. IPT is characterized by signs of nonspecific inflammation and fibrosis [5]. The pathogenesis of the lesion is unknown [1] but has been associated with various infectious processes, such as Epstein-Barr virus [6] or* Escherichia coli* [1].

We present here a unique case of pulmonary invasion by a liver IPT, followed by identification of* E. coli* in the sputum. This strengthens the association between* E. coli* and IPT.

## 2. Case Presentation

The patient was a 67-year-old African-American male veteran with a history of diabetes mellitus type 2 (hemoglobin A1c 6.6%) who presented with a fever of 39.5 and tachycardia to 115 following a routine outpatient CT scan for a liver mass that was discovered about a month earlier on ultrasound.

The patient had recently been treated empirically for a urinary tract infection with ciprofloxacin for seven days with symptomatic improvement. During the week prior to admission, he had been experiencing shaking chills and fevers, as well as a 4.5 kg loss due to anorexia and nausea without vomiting.

His past medical history was significant for a remote history of prostate cancer, treated with brachytherapy and a thyroid nodule found on thyroid ultrasound. He had not traveled outside the United States since serving in Vietnam.

**Figure 1:**
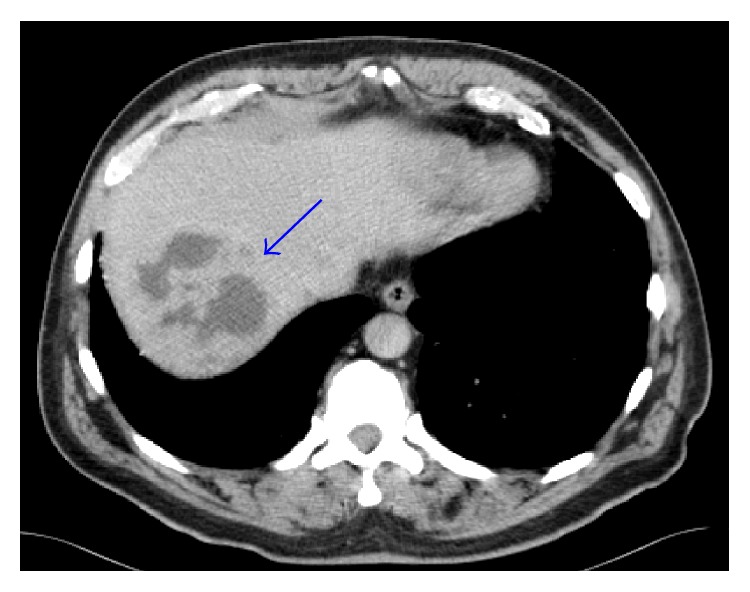
Loculated liver mass, as soon on admission CT.

Physical exam on admission was significant for fever and tachycardia, but otherwise, the patient appeared well, and the abdominal exam did not show tenderness or organomegaly. Liver function tests were within normal limits, and hepatitis B and C serologies were negative. Admission CT scan showed a multiloculated cystic mass within the liver (Figure 1). The differential diagnoses included primary and metastatic cancer, hepatic abscess, and parasite infection. Other much smaller low-density lesions were seen throughout the liver as well. There was also a nonocclusive thrombus in the portal vein, a mildly prominent right infrahilar lymph node measuring 1 cm, and bilateral renal cysts.

MRI with contrast was performed to further characterize the mass, but the differential diagnosis remained broad. The portal vein thrombus was observed again.

**Figure 2:**
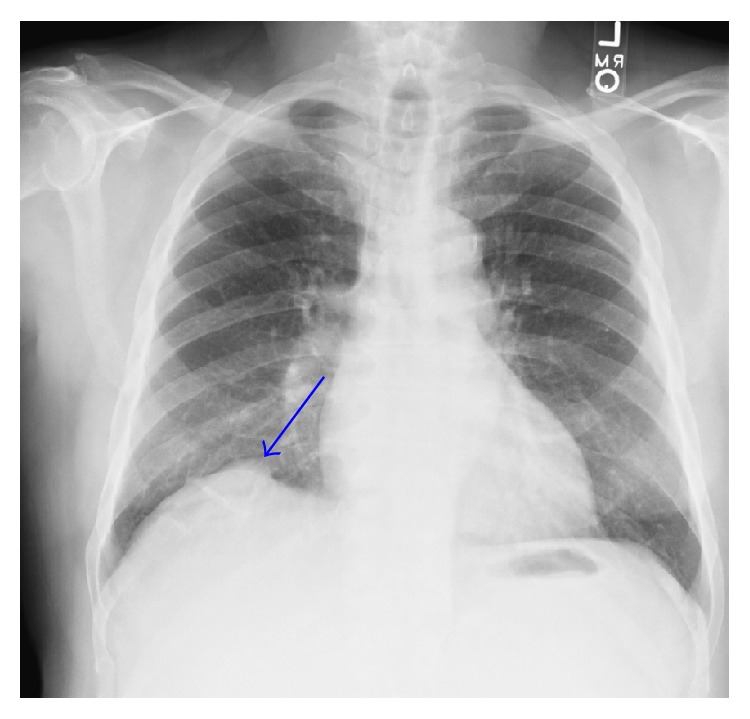
Chest X-ray illustrating the initial pulmonary infiltration of the liver mass (arrow).

**Figure 3:**
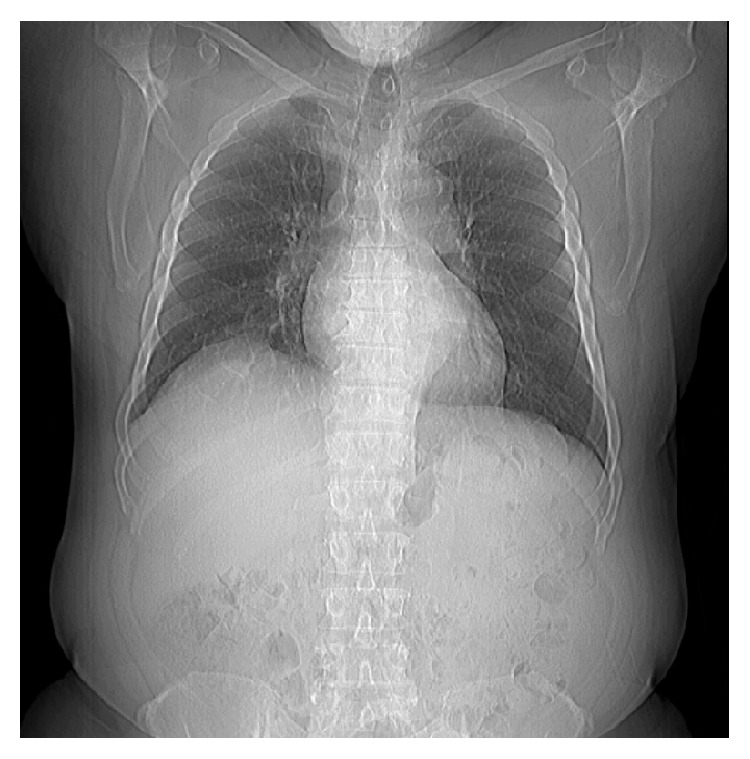
Admission CT scan did not show pulmonary infiltration.

The patient's fever had resolved shortly after admission, but he then again developed a fever up to 39.2 three days after admission. At that point, he was pancultured and started on vancomycin and piperacillin-tazobactam. Results of the sputum culture are shown in Table 1; other cultures and work-up were negative. Chest X-ray showed slight expansion of the liver mass into the lungs (Figure 2), which was not observed on the admission CT (see scout image, Figure 3).

**Figure 4:**
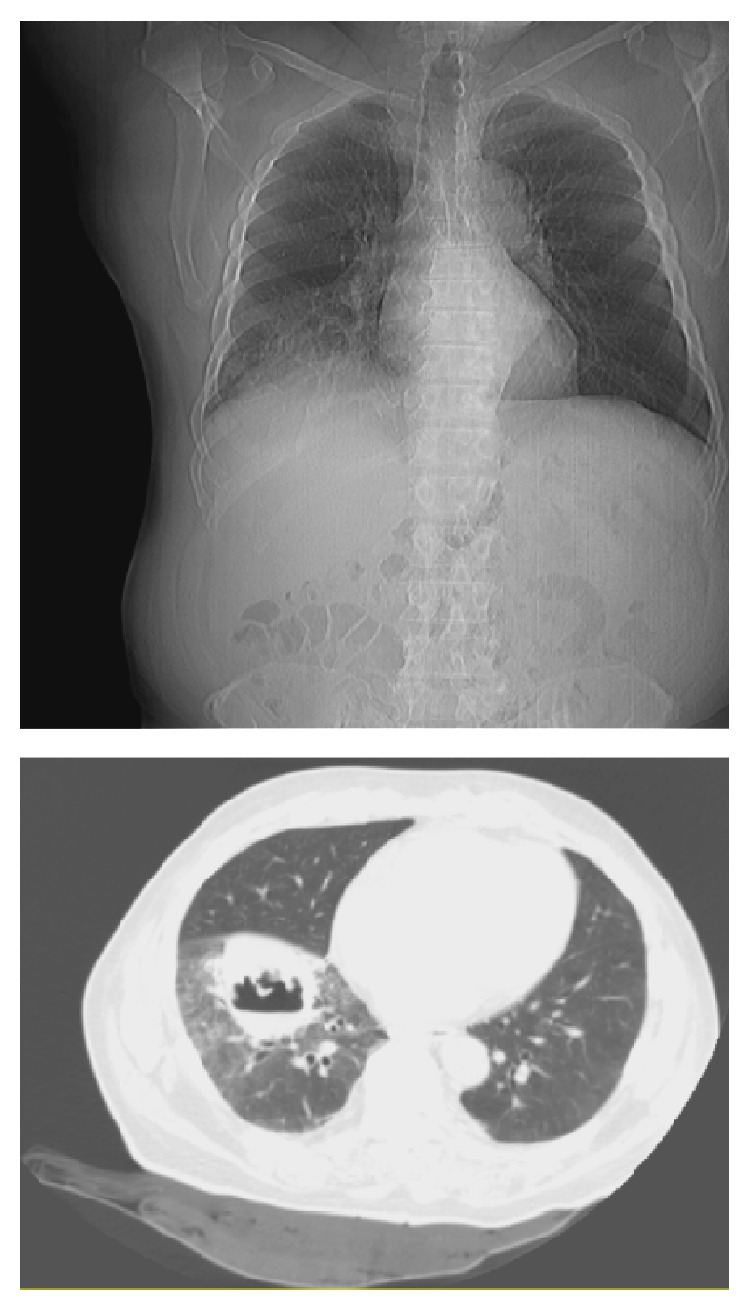
Rapid, major pulmonary infiltration by day six of admission.

The patient underwent a CT-guided biopsy of the liver lesion six days after the initial fever. On CT imaging taken for the biopsy, the lesion was noted to have spread, creating a cavitary lesion in the right lung (Figure 4). An aspirate of the cystic components did not show any sign of infection or malignancy on cytology. A PET scan was ordered but did not help narrow the differential diagnosis.

Due to the equivocal result of the first biopsy, a second biopsy was performed to obtain tissue from the solid portion of the mass. Pathology eventually showed multiple foci liver parenchymal replacement by an inflammatory and spindle cell infiltrate. Immunohistochemical stains showed the infiltrate to be positive for CD68. Pancytokeratin staining highlighted the entrapped hepatocytes and ductular structures with CK7, a marker for epithelium. CD117 (receptor tyrosine kinase associated with cancer) and S100 (associated with neural crest cells and some cancers) were negative; nonspecific staining was noted with DOG1 (tumor repressor). These findings were consistent with inflammatory pseudotumor.

The patient was discharged on intravenous ertapenem for three weeks and then transitioned to oral trimethoprim-sulfamethoxazole, to cover the* E. coli* found in the sputum. Follow-up ultrasound imaging showed a gradual decrease in the size of the lesion.

## 3. Discussion

This patient had an inflammatory pseudotumor of the liver with radiographic evidence of the process of infiltration of the right lung. Soon after this process was observed on imaging,* E. coli* was cultured from the sputum, further strengthening the association between* E. coli* and IPT.

IPT is a rare condition that can affect any organ but most commonly affects the lungs and the liver [5]. The symptoms and signs of inflammatory pseudotumor are not specific, which makes diagnosis challenging [2]. Intermittent fever [7] and weight loss [4] have been reported in previous cases of IPT of the liver and were consistent with the findings in this patient.

On imaging, the CT scans showed an irregular, loculated mass, with at least partial blockage of the portal vein. Similar findings have previously been reported for IPT of the liver [4, 5, 7]. Imaging findings were repeatedly equivocal, suggestive of either a malignancy or an abscess, but not typical for either one, which is also consistent with previous reports [4]. The remainder of the liver appeared normal on liver imaging, without radiographic or pathologic evidence of cirrhosis. There was no biochemical evidence of hepatitis, and liver function tests were unremarkable, also consistent with previous reports [8].

**Figure 5:**
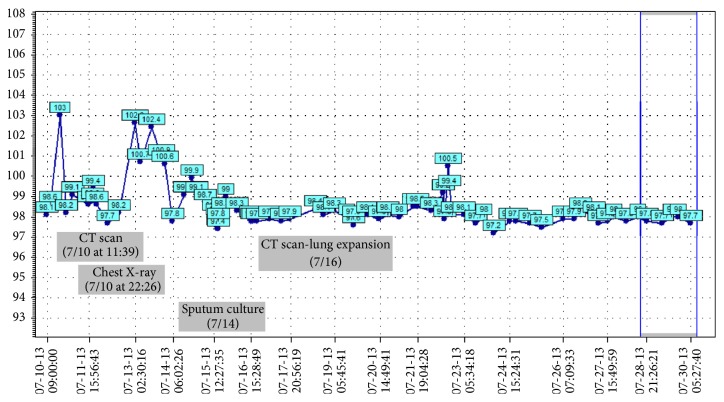
Sputum culture showed *E. coli* just after a fever spike, afterwhich the liver mass was found to have expanded into the lungs.

One unusual feature in this case was our ability to identify* E. coli*, which has been associated with IPT in a previous case report [8, 9], as a possible causal organism.* E. coli* was cultured from the patient's sputum, corresponding temporally to when the mass eroded through the right diaphragm and invaded the lungs (Figure 5). Other sputum cultures were negative, as were blood and urine cultures. Since* E. coli* is an uncommon organism to be isolated from the sputum, we believe that we can attribute the presence of this organism in the sputum to the infiltration of the IPT into the lungs from the liver. Treatment of the* E. coli* infection with intravenous followed by oral antibiotics caused the mass to shrink, reinforcing the idea that the infection was the source of the mass. However, further research, especially basic science research, is needed to determine whether there is a causal relationship between the IPT and* E. coli*.

Treatment of IPT has been somewhat controversial [10], with debate as to whether surgical or medical management is most appropriate. Treatment with antibiotics has been successful in some previous cases [4, 11]. The current case report supports treatment with antibiotics when the IPT is identified in the liver and is associated with fever and leukocytosis. These inflammatory pseudotumors may represent infectious processes due to displaced intestinal flora.

## 4. Conclusion

*Escherichia coli* infection in the presence of a rapidly changing mass can be a clue to the diagnosis of inflammatory pseudotumor.

## Figures and Tables

**Table 1:** Susceptibility profile of *Escherichia coli* cultured from sputum.

	Susceptible	Interpretation
	(*μ*g/mL)	
Amikacin	8	Susceptible
Ampicillin	≥32	Resistant
Cefazolin	≥64	Resistant
Ciprofloxacin	≥4	Resistant
Trimethoprim/sulfamethoxazole	≤20	Susceptible
Gentamicin	≥16	Resistant
Imipenem	≤1	Susceptible
Ampicillin/sulbactam	≥32	Resistant
Ertapenem	≤0.5	Susceptible
Cefepime	≤1	Susceptible
Piperacillin/tazobactam	≥128	Resistant
